# Determinants of sub-optimal glycemic control among patients enrolled in a medicine dispensing programme in KwaZulu-Natal: A cohort study, 2018–2021

**DOI:** 10.4102/phcfm.v16i1.4336

**Published:** 2024-05-31

**Authors:** Leigh C. Johnston, Patrick Ngassa Piotie, Innocent Maposa, Sandhya Singh, Lazarus Kuonza, Alex De Voux

**Affiliations:** 1 South African Field Epidemiology Training Program, National Institute for Communicable Disease, A Division of the National Health Laboratory Service, Johannesburg, South Africa; 2 Division of Epidemiology and Biostatistics, School of Public Health, University of the Witwatersrand, Johannesburg, South Africa; 3 Non-Communicable Disease Directorate, National Department of Health, Pretoria, South Africa; 4 Diabetes Research Centre, School of Health Systems and Public Health, University of Pretoria, Pretoria, South Africa; 5 Division of Epidemiology and Biostatistics, School of Public Health, Faculty of Health Sciences, University of Cape Town, Cape Town, South Africa

**Keywords:** CCMDD programme, glucose control, survival analysis, type 2 diabetes, eThekwini, glycaemic control

## Abstract

**Background:**

The Central Chronic Medicines Dispensing and Distribution (CCMDD) programme facilitates clinically stable patients to collect their chronic medication from community-based pick-up points.

**Aim:**

We determined baseline glycaemic control and rates and predictors of becoming sub-optimally controlled for type 2 diabetes mellitus (T2DM) CCMDD-enrolled patients.

**Setting:**

The setting of the study was eThekwini, KwaZulu-Natal, South Africa.

**Methods:**

We performed a cohort study (April 2018- December 2021). We linked T2DM CCMDD-enrolled patients to glycated haemoglobin (HbA1c) data from the National Health Laboratory Service. We selected patients optimally controlled at their baseline HbA1c, with ≥ 1 repeat-test available. We used Kaplan–Meier analysis to assess survival rates and extended Cox regression to determine associations between time to sub-optimal control (HbA1c > 7%) and predictors. Adjusted hazard ratios (aHRs), 95% confidence interval (CI), and *p*-values are reported.

**Results:**

Of the 41145 T2DM patients enrolled in the CCMDD programme, 7960 (19%) had a HbA1c result available. Twenty-seven percent (2147/7960) were optimally controlled at their baseline HbA1c. Of those controlled at baseline, 695 (32%) patients had a repeat test available, with 35% (242/695) changing to sub-optimal status. The HbA1c testing frequency as per national guidelines was associated with a lower hazard of sub-optimal glycaemic control (aHR: 0.46; 95% CI: 0.24–0.91; *p*-value = 0.024). Patients prescribed dual-therapy had a higher hazard of sub-optimal glycaemic control (aHR: 1.50; 95% CI: 1.16–1.95; *p*-value = 0.002) versus monotherapy.

**Conclusions:**

The HbA1c monitoring, in-line with testing frequency guidelines, is needed to alert the CCMDD programme of patients who become ineligible for enrolment. Patients receiving dual-therapy require special consideration.

**Contribution:**

Addressing identified shortfalls can assist programme implementation.

## Introduction

Diabetes mellitus is the second leading natural cause of death in South Africa (SA),^1^ affecting an estimated 4.2 million people.^2^ Ninety per cent of people living with diabetes have type 2 diabetes mellitus (T2DM), considered a preventable and potentially reversible condition.^2,3^ However, prevalence has continued to rise, disproportionately affecting women.^1^ The growing trend has been fuelled by lifestyle factors (e.g. urbanisation, increased consumption of processed foods, reduced physical activity, and obesity) as well as social and commercial determinants of health.^2,3^

Because of the chronic nature of the disease, people living with T2DM (PLWT2DM) need to monitor their glycaemic levels regularly, thus continuously assessing their disease progression and preventing T2DM-related complications.^2,3^ Type 2 diabetes mellitus-related complications include macrovascular diseases (i.e. cardiovascular disease [CVD] or stroke) and microvascular diseases (i.e. retinopathy, neuropathy, and nephropathy).^4^ Sub-optimal or poor glycaemic control has been linked to a 37% increased risk of microvascular diseases^5^ and a 2- to 4-fold increased risk of macrovascular diseases.^6^

Various tests exist to monitor glycaemic control, including fasting plasma glucose (FPG) and glycated haemoglobin (HbA1c) tests. Fasting plasma glucose is an indicator of short-term glycaemic levels, and is in constant flux, as it is dependent on factors such as food consumed, stress or physical activity.^7^ HbA1c is a longer-term indicator of glycaemic control, over 2–3 months,^7^ and is considered the gold standard for glycaemic monitoring by the International Diabetes Federation (IDF).^2^ When using HbA1c, sub-optimal glycaemic control, is defined by test readings of > 7%.^3,8^

Current guidelines by the Society for Endocrinology, Metabolism and Diabetes South Africa (SEMDSA) recommend HbA1c monitoring on a three-monthly basis if control is sub-optimal and a six-monthly basis if control is optimal.^3^ The standard treatment guidelines (STG) for primary health care (PHC) in SA recommend three- to six-monthly HbA1c testing if sub-optimally controlled (HbA1c > 7%) and 12-monthly testing if optimally controlled (HbA1c ≤ 7%).^8^ Those with sub-optimal glycaemic control require more frequent monitoring, to allow for early corrective actions to achieve optimal control. Early corrective actions help prevent T2DM-related complications, which impose considerate costs to the public health service and social and economic costs to PLWT2DM.^2^

Considering the magnitude of the public health problem posed by non-communicable diseases (NCD) including diabetes, the SA National Department of Health (NDoH) launched the National Strategic Plan (NSP) for the Prevention and Control of Non-Communicable Diseases 2022–2027, in May 2022.^9^ The NSP aimed, among others, to ensure that 50% of those receiving T2DM treatment, achieve glycaemic control by 2030.^9^

Underpinning the NSP, are existing policies, such as the Primary Health Care Re-engineering policy and, the Integrated Chronic Disease Management approach. These include developing, and mobilising existing community-based services (e.g. support groups and community-based medication delivery mechanisms), to manage NCD patients at the community level.^10,11^ The Central Chronic Medicines Dispensing and Distribution (CCMDD) programme is one such initiative and was launched in 2014 by the NDoH.^12^ The CCMDD aims to allow people living with HIV and/or NCDs, who are considered stable for these conditions, to collect their chronic medications from community-based pick-up points, such as independent pharmacies, adherence clubs, and smart lockers every second month.^11,12^ This resulted in task-shifting the dispensing and collection of chronic medication from congested clinics, and relieving overburdened staff, and improving quality of care.^12,13^ Additionally, the programme addresses commonly cited barriers to accessibility and adherence to medication, such as reducing transportation costs for patients, and negating long waiting times and medication stock-outs at clinics.^12,13^

Type 2 diabetes mellitus patients are eligible to be enrolled into CCMDD if they are 18 years or older, able to give consent, and considered stable.^12^ Being stable on medication was defined as having two consecutive normal FPG readings of less than 7 mmol/L, being on medication for more than 6 months, and having no changes in medication in the last year.^12^ Non-stable patients with T2DM, as per the STG, attend clinics quarterly,^8^ while non-enrolled stable patients^8^ and CCMDD-enrolled stable patients attend clinics biannually for follow-up and for prescription renewal.^12^

KwaZulu-Natal (KZN), and the district of eThekwini, in particular, have been highlighted by recent research as an area of particular concern for their diabetes disease burden, with eThekwini representing 45%–55% of newly diagnosed cases in the province.^14,15^ To our knowledge, there are no prior studies estimating the numbers of PLWT2DM in eThekwini, KZN who are receiving medication and are adequately controlled since the launch of the NSP, nor are there studies within the CCMDD, addressing changes in glycaemic control over time. As the CCMDD does not collect clinical glycaemic control data, merging and enhancing the CCMDD patient information data with laboratory data housed in the central data warehouse (CDW) of the National Health Laboratory Service (NHLS) clinic provides a unique opportunity to follow a cohort of patients retrospectively, accounting for a variation in HbA1c over time. This cohort study builds on a previous study from Gauteng province in South Africa, to determine glycaemic control within the CCMDD programme.^16^ In this article, we determined the proportions of CCMDD-enrolled patients achieving optimal glycaemic control and looked at factors associated with an increased hazard for developing sub-optimal control. Identifying, understanding and integrating these factors or vulnerabilities into diabetes care would prevent diabetes-related morbidity and mortality and reduce expenses related to uncontrolled T2DM.

## Research methods and design

### Study design

We conducted a retrospective cohort study to describe HbA1c test results extracted from the NHLS CDW, for all T2DM CCMDD-enrolled patients receiving oral hypoglycaemic medications through the programme between April 2018 and December 2021 in eThekwini, KZN, SA.

### Study setting

The study took place in the public health sector in the district of eThekwini in KZN, SA.

### Data sources

We sourced our data from the CCMDD and NHLS CDW. The CCMDD has achieved a reach of 88%, with 94.6% of facilities in the districts represented.^12^ As such, CCMDD reaches eight community health clinics, 99 clinics, and 13 hospitals within eThekwini. The NHLS is the sole provider of laboratory services for the public health system, serving 80% of the population.^17^

### Study participants

The study participants were all T2DM adult (≥ 18 years) male and female patients enrolled in the eThekwini CCMDD over the study period (April 2018–December 2021). As this study used secondary data, no sampling was done. We selected those with non-missing HbA1c data from the NHLS CDW. We allowed a 6-month window, for the HbA1c reading from the clinical visit preceding the patient’s first CCMDD prescription date, and the last CCMDD prescription date, to be included (October 2017 and June 2022). For the survival analysis, we selected those optimally controlled at their first HbA1c measure and with a repeat HbA1c test/s available between October 2017 and June 2022.

### Variables and data management

Central Chronic Medicines Dispensing and Distribution data were provided as an Excel spreadsheet and were exported to STATA version 17 for cleaning and analysis. The CCMDD data included date of birth, sex, healthcare facility, T2DM-related complications, and details of the first and last prescription (medication and prescription dates). The data were checked for errors, inconsistencies, outliers and missing data. Any duplicate participants and duplicate observations per participant were identified and removed. Data were transformed from a long to a wide format. These data were sent as an Excel spreadsheet to the NHLS gatekeepers, who matched Hba1c test results to the CCMDD line list using the national identity number. The NHLS HbA1c data included the date of the specimen, the date of the laboratory test, and the test name and value. These data were sent back to the primary investigator. The variables for HbA1c test date, the HbA1c result, and the unique patient ID, were used to determine a duplicate HbA1c observation. Data variables were unstrung and formatted as numerical, date values or encoded as categorical variables, as was appropriate.

#### Outcome variable

We used HbA1c values from the NHLS merge, which was a continuous measure. We created a binary variable for optimal and sub-optimal control. This study defined glycaemic control as optimal if the HbA1c reading was 7% or less and as sub-optimal for values above 7%. This is per the 2017 SEMDSA guidelines and the 2020 South African STG.^3,8^ We defined the baseline HbA1c value as the first recorded NHLS value within the study period. For the survival analysis, only those optimally controlled at their first reading in the study period were used, and time-to-failure was calculated for the first change of status to sub-optimal control.

#### Demographic characteristics

Sex was sourced from the CCMDD data and was coded as either male or female. We calculated age using the ‘datediff’ command to subtract the HbA1c sample collection date from the patient’s date of birth. Once calculated, we categorised age in years into four levels, as follows: 18–39, 40–59, 60–79, and 80 or over. We used these categories as the age data were skewed suggesting nonlinearity. We included the categories 18–39 and 80 or over, despite the relatively fewer values to isolate the aberrant median HbA1c values in these categories.

#### Diabetes severity

To manage diabetes, the STG and Essential Medication List (EML) of SA advise lifestyle modification, followed by progressive pharmacological therapies; starting with monotherapy (i.e. metformin) and are up-scaled to dual-therapy (i.e. metformin and a sulphonylurea agent, namely, glibenclamide or glimepiride), and finally to insulin, if a patient is still sub-optimally controlled or if their disease progresses.^8^ We used the ‘strops’ function to identify these medications used for diabetes management. We then used ‘egen’ function to total tags for metformin, glibenclamide and glimepiride for each unique patient ID, to create a categorical variable with three levels, as follows: monotherapy (one oral hypoglycaemic agent prescribed), dual-therapy (two hypoglycaemic agents prescribed) and triple-therapy (three hypoglycaemic agents prescribed) in the same prescription. As the patient’s prescriptions changed over time, we used the following coding strategy. If the patient used two medications at any point over the study period, or changed from monotherapy to dual-therapy, they were placed in the dual-therapy category. Those that only ever used one oral agent were placed in the monotherapy category. It is notable, that it is not within the recommendations to prescribe three oral hypoglycaemic agents to manage diabetes, so we include this category with only a few values, to describe these aberrant cases. Notably, insulin-using PLWT2DM are not eligible for the programme because of cold-chain procedures and the need for monthly clinical monitoring for titration.^18^

We used the ‘strops’ command in Stata, to identify facilities that were hospitals, and those that were PHC facilities (i.e. clinics or CHCs), based on the facility name. We created a variable for facility type, with two levels, namely hospital or PHC facility. We also used the ‘strops’ function to identify neuropathy or nephropathy T2DM-related complication data and created a binomial variable, indicating if there were no recorded complication, or if there was a recorded complication (i.e. neuropathy or nephropathy). Notably, diabetic retinopathy data were unavailable in either data source. As the study period was over 3 years, and T2DM-related complications and facility type did not vary over time, we used the baseline category in our coding strategy.

#### Quality of care

The SEMDSA clinical care guidelines recommend 3 months between HbA1c tests for those sub-optimally controlled, and 6 months between tests for those optimally controlled.^3^ We calculated adherence to the SEMDSA testing frequency guidelines for each patient by calculating the time between each HbA1c test and the test that preceded it with the Stata ‘datediff’ command. If the preceding test was optimal, we used an interval of 6 months or less to define adherence and an interval of more than 6 months to define non-adherence. If the preceding test was sub-optimal, we used an interval of 3 months or less to define adherence and an interval of more than 3 months to define non-adherence. We then summed tests that adhered to the recommended time interval for each patient, and divided this by the number of tests per patient minus the first test (i.e. *N* − 1). We subtracted the first test as it did not have a test preceding it. We considered a proportion of 1 as the patients adhering to the guideline for all of their tests (e.g. if a patient had three HbA1c tests, and two adhered to the recommended time interval, then their adherence rate was 2/ (3 − 1) = 1). Thus, the adherence variable was binomial.

#### Comorbidity

The CCMDD is used to distribute medication for other stable chronic conditions (e.g. hypertension [HPT], hyperlipidaemia, and HIV). We used ‘strops’ to identify the relevant conditions, to create the comorbidity variable: T2DM alone, T2DM and HIV, T2DM and dyslipidaemia, and multimorbidity (i.e. T2DM, HPT, HIV).

### Data analysis

STATA v17 was used to conduct the analysis. We used summary statistics to describe the HbA1c tests available, as well as the frequency and proportion of those with one, two, or more than three HbA1c tests performed over the study period. We also calculated the median and inter-quartile range (IQR) time interval between tests in months, median age and the median months between HbA1c test and CCMDD enrolment date. Medians and IQR were reported for all non-normally distributed continuous variables, on statistical (sktest and swilk) and graphical tests.

We summarised baseline patient characteristics (i.e. sex, age, type of facility, T2DM-related complications, type of oral hypoglycaemic therapy received and comorbidities) using frequencies and proportions for all categorical data. We summarised median HbA1c values across each covariate’s strata. Additionally, frequencies and proportions were reported by optimal and sub-optimal control across each covariate’s strata. The Chi-square test was used to assess the association between glycaemic control status (optimal vs. sub-optimal) and each covariate (i.e. sex, age, type of facility, T2DM-related complications, type of oral hypoglycaemic therapy received and comorbidities). A *p*-value less than 0.05 was considered statistically significant in the Chi-square test.

For those who had an optimal HbA1c test value at baseline, with at least one repeat test value available, we performed a survival analysis. The outcome of interest was years until sub-optimal glycaemic control. It was generated by using the date of the first sub-optimal event, in the study period. For those that remained optimally controlled to the end of the study period (i.e. those who were right censored), we used the last date of the study period (i.e. 30 June 2022). We calculated the median age (IQR), median HbA1c (IQR), median (IQR) months between HbA1c tests, median (IQR) months between the first available HbA1c test and the CCMDD enrolment date, median (IQR) number of tests per unique patient, and median (IQR) analysis time in years, by glycaemic control outcome. We summarised these patient characteristics using frequencies and proportions for sex, age, type of facility, T2DM-related complications, type of oral hypoglycaemic therapy received, and comorbidities.

Univariate analysis was conducted, using Kaplan–Meier curves to analyse the survival curves for the probability of maintaining optimal control by potential predictor variables (i.e. sex, age, type of facility, T2DM-related complications, type of oral hypoglycaemic therapy received and comorbidities). The log-rank test was used to compare survival curves across categorical variables’ strata. We summarised frequencies, sub-optimal event frequencies, and time at risk for each covariate’s (i.e. sex, age, type of facility, T2DM-related complications, type of oral hypoglycaemic therapy received, and comorbidities) strata. Incidence rates for developing sub-optimal control were calculated by dividing the number of sub-optimal events by the time at risk (in person-years), for each covariate’s strata. Incidence rates and 95% confidence interval (CI) were reported per 1000 person-years. We also assessed the 25th percentile of survival time in years (i.e. the analysis time where 25% of subjects have become sub-optimally controlled and 75% remain optimally controlled) for each covariate strata. Variables with a *p* < 0.25 were included in the multivariable Cox model. We tested interaction terms between all covariates; however, none were significant.

The variables sex, facility type, type of therapy, and adherence to SEMDSA guidelines for HbA1c testing frequency were included in the final model. The assumption of proportional hazards (PH) was not upheld in the global test because of the variable facility type; consequently, an extended Cox analysis, which is used for analysis where time-varying variables exist, was conducted. We summarised the co-efficient (95% CI) and aHR (95% CI) for the main model and for the time-varying coefficient model. We calculated a combined effect by adding the two models coefficients (i.e. 1- *ℯ*^(main model co-efficient+ time-varying co-efficient)^). A CI of 95% was used and a significance level of *p* < 0.05 was considered statistically significant for the Cox regression model tests.

### Ethical considerations

Ethical approval was obtained from the University of the Witwatersrand’s Medical Human Research Ethics Committee (HREC no. M220232). Data access approval was obtained from the relevant custodians in the CCMDD and NHLS.

## Results

### Participants

**FIGURE 1:**
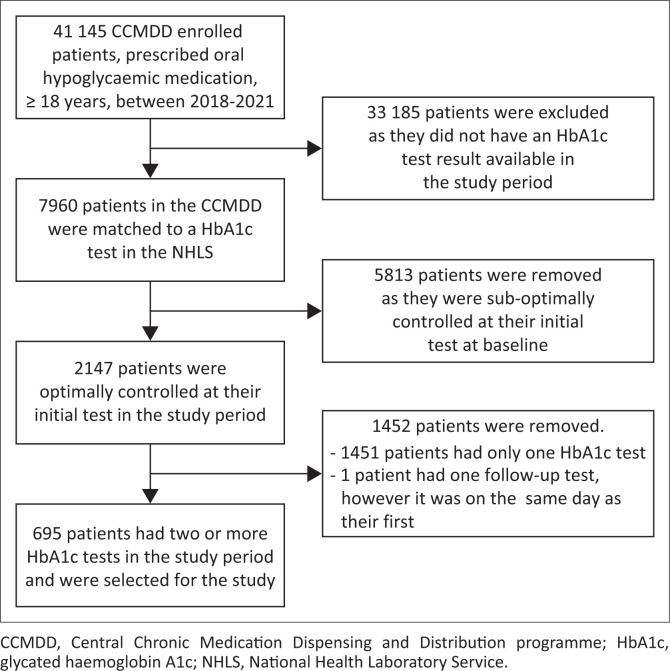
Flow chart of study population selection.

There were 41 145 CCMDD-enrolled patients in eThekwini, who were 18 years or older and receiving oral hypoglycaemic medication between 2018 and 2021 (Figure 1). Nineteen per cent (7960/41 145) had one or more HbA1c test result. Among patients with a HbA1c test available, 27% (2147/7960) were optimally controlled at baseline. Of these, 695 patients had one or more repeat HbA1c test result in the study period (Figure 1).

### Descriptive data

#### Baseline analysis results

For the 7960 CCMDD-enrolled patients, there were 12 102 HbA1c tests performed over the study period (Table 1). Of the 12 102 HbA1c test results, 66% (*n* = 7960) were first observations, 22% (*n* = 2646) were second observations, and 12% (*n* = 1496) were the third or more observations for a unique patient. The overall median interval between tests for those with more than one test available was 12 months (IQR: 8–18). The median age of patients was 60 years (IQR: 52–67) and the median HbA1c was 8.2% (IQR: 7.0–10.0).

**TABLE 1:** Description of HbA1c samples for the Central Chronic Medicines Dispensing and Distribution programme cohort receiving oral hypoglycaemic medication for type 2 diabetes mellitus, eThekwini, KZN, South Africa: 19 April 2018 – 30 December 2021 (*N* = 7960).

Characteristic	Number of HbA1c tests per patient											
	1			2			≥ 3†			Total		
	*N*	%	IQR	*N*	%	IQR	*N*	%	IQR	*N*	%	IQR
HbA1c samples	7960	65.8	-	2646	21.9	-	1496	12.4	-	12 102	100.0	-
Sub-optimally controlled	5984	75.2	-	1964	74.2	-	1181	78.9	-	9129	75.4	-
Median HbA1c result	8.1	-	7.0 to 10.0	8.1	-	6.9 to 9.9	8.4	-	7.1 to 10.1	8.2	-	7.0 to 10.0
Median months between 1st HbA1c test and CCMDD enrolment date	4	-	−8 to 18	12	-	−1 to 26	18	-	5 to 31	7	-	−6 to 22
Median months between HbA1c tests	-	-	-	12	-	8 to 19	9	-	6 to 13	12	-	8 to 18
Median age in years	60	-	52 to 67	60	-	52 to 67	60	-	52 to 67	60	-	52 to 67

CCMDD, Central Chronic Medicines Dispensing and Distribution; IQR, inter-quartile range.

† , Those with three or more observations were grouped.

Demographic characteristics of the 7960 patients with a baseline HbA1c are summarised by glycaemic control status (Table 2). Those who were excluded for being sub-optimally controlled at baseline differed from those that were included by sex, age, and type of therapy, and were similar by type of facility, T2DM-related complications, and comorbidity (Table 2).

**TABLE 2:** Baseline median HbA1c value by patient characteristic for the Central Chronic Medicines Dispensing and Distribution programme cohort receiving oral hypoglycaemic medication for **type 2 diabetes mellitus**, eThekwini, KZN, South Africa: 19 April 2018 – 30 December 2021 (*N* = 7960).

Characteristic	Total *N* = 7960 (100%)		Median HbA1c	IQR	Sub-optimal (HbA1c > 7%)‡		Optimal (HbA1c ≤ 7%)§		*p* †
	*N*	%			*n*	%	*n*	%	
**Sex**									< 0.001†***
Male	2608	33	7.9	6.8–9.8	1816	31	792	37	-
Female	5352	67	8.2	7.0–10.1	3997	69	1355	63	-
**Age category (years)**									< 0.001†***
≤ 39 years	311	4	8.5	7.1–11	234	4	77	4	-
40–59 years	3566	45	8.6	7.2–10.5	2759	47	807	38	-
60–79 years	3831	48	7.8	6.9–9.5	2692	46	1139	53	-
≥ 80 years	252	3	7.1	6.5–8.1	128	2	124	6	-
**Type of facility**									0.92†
PHC facility	5195	65	8.1	6.9–9.9	3792	65	1403	65	-
Hospital	2765	35	8.2	7.0–10.1	2021	35	744	35	-
**T2DM-related complication**									0.23†
Neuropathy or nephropathy	4135	52	8.1	6.9–9.9	2996	52	1139	53	-
None recorded	3825	48	8.2	7.0–10.1	2817	48	1008	47	-
**Type of therapy**									< 0.001†***
Monotherapy	4418	56	7.6	6.7–9.4	2870	49	1548	72	-
Dual-therapy	3538	44	8.8	7.4–10.6	2939	51	599	28	-
Triple-therapy	4	0	9.8	9.3–10.1	4	0	0	0	-
**Comorbidity**									0.097†
T2DM alone	1175	15	8.4	7.0–10.3	880	15	295	14	-
T2DM and HIV	450	6	8.6	7.0–10.5	335	6	115	5	-
T2DM and HPT	5608	70	8.1	7.0–9.9	4094	70	1514	71	-
T2DM, HIV and HPT	704	9	8.0	6.8–10.1	487	8	217	10	-
T2DM and dyslipidaemia	23	0	8.5	6.8–10.4	17	0	6	0	-

T2DM, type 2 diabetes mellitus; PHC, primary health care; HIV, human immunodeficiency virus; HPT, hypertension; IQR, inter-quartile range.

† , *p*-value for the chi-square test for sub-optimal versus optimal;

‡ , *N* = 5813 (73%);

§ , *N* = 2147 (27%).

Significance level (*p*-value):

*** , *p* < 0.001. The p-values in table 2 are either not significant or are significant at < 0.001 level. Please edit appropriately.

#### Survival analysis results

Among the 695 study participants who had an optimal baseline HbA1c test result, and with one or more repeat HbA1c test result (Table 3), the median age was 61 years (IQR: 53–69). The overall median interval between HbA1c tests was 13 months (95% CI: 10 – 19) and was similar for those optimally (12 months; 95% CI: 10 – 19) and sub-optimally controlled (13; 95% CI: 10 – 20) (Table 3). The median time between the first HbA1c test and the patient’s CCMDD enrolment date was 3 months before enrolment (95% CI: 13 months prior – 10 months post) (Table 3). The median number of HbA1c tests per patient was 2 (IQR: 2–2), with 2 as a minimum and 13 as a maximum. The median time in the study was 4.7 years for those who remained optimally controlled and was 3.2 years for those who became sub-optimally controlled (Table 3). Of the 695 patients, 23 HbA1c tests were in the period of the level 4–5 coronavirus disease 2019 (COVID-19) lockdown in SA (i.e. 27 March 2020 – 01 June 2020). The median time between test during the lockdown period was 11.8 (95% CI: 6.4–14.9) months versus 12.1 (95% CI: 8.9–20.1) months in lesser level or non-lockdown periods.

**TABLE 3:** Descriptive characteristics of the Central Chronic Medicines Dispensing and Distribution cohort with ≥ 1 repeat HbA1c test value available eThekwini, KwaZulu-Natal, South Africa: 19 April 2018 – 30 December 2021 (*N* = 695).

Characteristic	Total (*N* = 695)			Sub-optimal event (*N* = 242)			Remained optimal (*N* = 453)		
	Median	IQR	min–max	Median	IQR	min–max	Median	IQR	min–max
Age	61	53 to 69	-	59	51 to 68	-	61	54 to 69	-
HbA1c	6.7	6.1 to 7.3	-	7.5	7.2 to 8.6	-	6.3	6.0 to 6.6	-
Months between HbA1c tests	13	10 to 19	-	13	10 to 20	-	12	10 to 19	-
Months between CCMDD enrolment date and baseline HbA1c test date	−3	−13 to 10	-	−4	−15 to 6	-	−2	−13 to 11	-
Number HbA1c tests	2	2–2	2–13	2	2–2	2–6	2	2–2	2–13
Analysis time in years	4.7	3.8–4.7	0.4–4.7	3.2	2.0–3.9	0.4–4.7	4.7	4.7–4.7	4.7–4.7

CCMDD, Central Chronic Medicines Dispensing and Distribution; IQR, inter-quartile range.

Additionally, the majority attended primary health care facilities (71%; 496/695) and were prescribed monotherapy (70%; 484/695) (Table 4). The most common comorbidity was hypertension (71%; 490/695), and 54% of the participants (377/695) having a T2DM-related complication of neuropathy or nephropathy (Table 4).

**TABLE 4:** Univariate survival analysis (Kaplan–Meier and log-rank test values) across potential predictor’s strata for the Central Chronic Medicines Dispensing and Distribution programme cohort, eThekwini, KwaZulu-Natal, South Africa: 19 April 2018–30 December 2021 (*N* = 695).

Characteristic	Number of cases	Number of sub-optimal events	Time at risk (years)	Incidence rate of sub-optimal glycaemic control per 1000 person-years	95% CI	25th percentile of survival time (years)†	*p*-values
**Sex**							0.1217§
Male	248	95	612.73	155.04	126.80–189.58	2.61	-
Female	447	147	1155.89	127.17	108.19–149.49	3.16	-
**Age (years)**							0.3238
17–39	24	10	68.44	146.11	78.61–271.55	3.62	-
40–59	298	113	740.70	152.60	126.87–183.45	2.69	-
60–79	333	110	857.87	128.22	106.37–154.57	2.86	-
≥ 80	40	9	101.60	88.58	46.09–170.25	3.53	-
**Type of facility**							0.0257*§
PHC facility	496	180	1180.09	152.53	131.80–176.52	2.91	-
Hospital	199	62	588.52	105.35	82.13–135.12	3.03	-
**T2DM-related complication**							0.3447
Nephropathy or neuropathy	377	125	969.34	128.95	108.22–153.66	2.71	-
None recorded	318	117	799.27	146.38	122.12–175.46	3.18	-
**Type of therapy**							0.0006**§
Monotherapy	484	150	1269.52	118.15	100.68–138.66	3.41	-
Dual-therapy	211	92	499.10	184.33	150.27–226.12	2.12	-
**Comorbidity**							0.8315
T2DM alone	86	28	217.91	128.50	88.72–186.10	3.18	-
T2DM and HIV	39	16	101.36	157.86	96.71–257.67	1.92	-
T2DM and HPT	490	168	1256.94	133.66	114.90–155.48	2.88	-
T2DM, HIV and HPT	80	30	192.41	155.91	109.01–222.99	3.34	-
**Months enrolled in CCMDD**							0.7485
≤ 6 months	230	79	609.44	129.63	103.98–161.61	2.74	-
7–12 months	69	23	180.80	127.21	84.54–191.43	3.60	-
≥ 13 months	388	138	957.67	144.10	121.96–170.26	2.71	-
**HbA1c testing frequency adheres to SEMDSA guideline** ‡							0.0064**§
Yes	63	9	150.34	59.86	31.15–115.05	4.44	-
No	632	233	1618.28	143.98	126.63–163.71	2.86	-
**Total**	**695**	**242**	**1768.62**	**136.83**	-	**2.88**	-

PHC, primary health care; T2DM, type two diabetes mellitus; HIV, human immunodeficiency virus; HPT, hypertension; CCMDD, Central Chronic Medicines Dispensing and Distribution; CI, confidence interval; SEMDSA, Society for Endocrinology, Metabolism and Diabetes South Africa.

† , The analysis time where 25% of subjects have become sub-optimally controlled and 75% remain optimally controlled;

‡ , SEMDSA clinical care guidelines recommend 3 months between HbA1c tests for those sub-optimally controlled, and 6 months between tests for those optimally controlled;

§ , Log-rank test of equality across strata, with *p*-values below 0.25 for the predictors, will be included as potential candidates for the final Cox model.

### Univariate results

#### Demographic information

**FIGURE 2:**
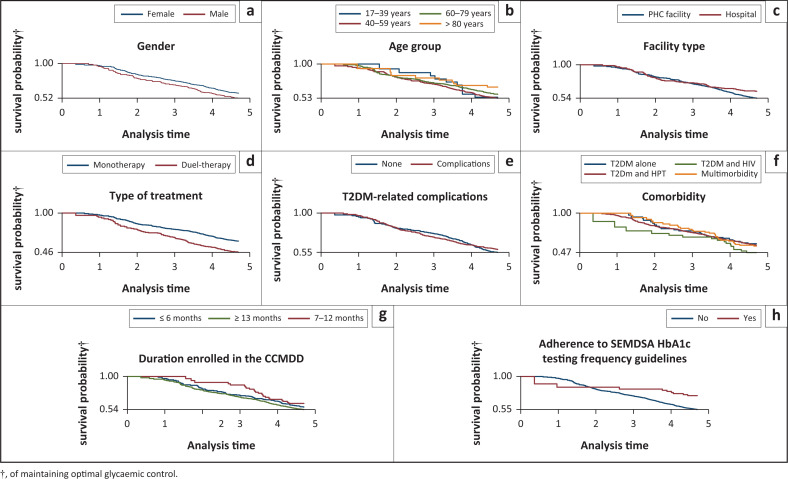
Kaplan–Meier survival curves for the probability of maintaining optimal glycaemic control, by potential predictors (a) gender, (b) age group, (c) facility type, (d) type of treatment, (e) T2DM-related complications, (f) co-morbidity, (g) duration enrolled in the CCMDD, (h) adherence to SEMDSA HbA1c testing frequency guidelines), for the Central Chronic Medicines Dispensing and Distribution programme cohort, eThekwini, KwaZulu-Natal, South Africa: 19 April 2018–30 December 2021 (*N* = 695).

The incidence rate for developing sub-optimal control over time was higher for male patients (155.04 per 1000 person-years) than for female patients (127.17 per 1000 person-years); however, it was not significant (*p* = 0.12) (Table 4 and Figure 2). Age was also not significantly associated with developing sub-optimal glycaemic control (*p* = 0.32), with higher incidence rates for younger age groups (146.11 and 152.60 per 1000 person-years among those aged ≤ 39 years and those aged 40–59 years, respectively); and lower incidence rates for those aged 60–79 years (128.22 per 1000 person-years) and those aged ≥ 80 years (88.58 per 1000 per years) (Table 4 and Figure 2).

#### Diabetes severity

Patients attending PHC facilities had a significantly higher incidence rate of developing sub-optimal control (152.53 per 1000 person-years), compared to those attending hospital facilities (105.35 per 1000 person-years; *p* = 0.03) (Table 4 and Figure 2). Compared to patients with no reported diabetes-related complications, patients with nephropathy and neuropathy had a lower incidence of sub-optimal control (128.95 vs. 146.38 per 1000 person-years; *p* = 0.35); however, it was not statistically significant (Table 4 and Figure 2). Patients on dual-therapy had a significantly higher incidence of sub-optimal glycaemic control, at 184.33 per 1000 person-years, compared to those who only used one oral medication at 118.15 per 1000 person-years (*p* = 0.00) (Table 4 and Figure 2).

#### Quality of care

The incidence rate of sub-optimal glycaemic control was significantly higher for those who did not have a follow-up HbA1c test within SEMDSA-recommended guidelines (i.e. within 3 months for those sub-optimally controlled and within six months for those optimally controlled) (143.98 per 1000 person-years) compared to those who did have a follow-up test within the SEMDSA-recommended time frame (59.86 per 1000 person-years, *p* = 0.01) (Table 4 and Figure 2). Those enrolled in the CCMDD between 7 and 12 months had lower incidence rates of sub-optimal control than those enrolled for < 7 or > 12 months; however, this was not a significant finding (*p* = 0.75).

#### Comorbidities

Patients with T2DM alone and with HPT comorbidity had lower incidence rates (128.50 and 133.66 per 1000 person-years, respectively), than those with multimorbidity (i.e. T2DM, HPT, and HIV), and those with HIV comorbidity (155.91 and 157.86 per 1000 person-years, respectively). However, these results were not statistically significant (*p* = 0.83) (Table 4 and Figure 2).

### Multivariable regression results

Predictors from the log-rank test of equality, with *p*-values below 0.25, were sex, facility type, type of therapy, and adherence to SEMDSA guidelines for HbA1c testing frequency, and were included in the final model.

#### Demographic information

When holding other variables constant, male patients had a 21 times higher hazard than female patients for developing suboptimal control over the study period; however, this finding was not statistically significant (*p* = 0.14) (Table 5).

**TABLE 5:** Multivariable Cox regression model for factors associated with developing sub-optimal glycaemic control for the Central Chronic Medicines Dispensing and Distribution programme cohort, eThekwini, KwaZulu-Natal, South Africa: 19 April 2018–30 December 2021 (*N* = 695).

Characteristic	Co-efficient	95% CI	Adjusted hazard ratio	95% CI	*p*
**Main model**					
**Sex**					
Male	0.194	−0.064 to 0.453	1.214	0.938 to 1.572	0.141
Female	1	-	1	-	-
**Facility type**					
PHC	1	-	1	-	-
Hospital	0.372	−0.276 to 1.020	1.450	0.759 to 2.771	0.261
**Type of therapy**					
Monotherapy	1	-	1	-	-
Dual-therapy	0.407	0.147 to 0.668	1.503	1.158 to 1.950	0.002**
**HbA1c testing frequency adheres to SEMDSA guideline** †					
Yes	−0.770	−1.441 to -0.100	0.463	0.237 to 0.905	0.024*
No	1	-	1	-	-
**Time-varying coefficient model**					
**Facility type × ln(t)**					
PHC	1	-	1	-	-
Hospital	−0.690	−1.313 to -0.065	0.502	0.269 to 0.937	0.031*

CI, confidence interval; PHC, primary health care; SEMDSA, Society for Endocrinology, Metabolism and Diabetes South Africa.

† , SEMDSA clinical care guidelines recommend 3 months between HbA1c tests for those sub-optimally controlled, and 6 months between tests for those optimally controlled.

Significance level (p-value):

* , *p* < 0.05;

** , *p* < 0.01.

#### Diabetes severity

The adjusted hazard for those attending hospitals, in the main effects model, was 45% higher than those attending PHCs (*p* = 0.26). However, on treating facility type as a time-varying covariate, by interacting its effect with the natural log of time, this hazard decreases by 50% for each unit increase in the natural log of time (*p* = 0.03). Thus, the combined effect is that of a 27% reduction in risk of developing sub-optimal control for those attending hospitals compared to those attending PHC facilities over the study period. The adjusted hazard for those using dual-therapy was 50 times higher than the hazard among those using monotherapy (*p* = 0.00) (Table 5).

#### Quality of care

Patients who had their HbA1c tests according to the SEMDSA-recommended guidelines (i.e. within 3 months for those sub-optimally controlled and within six months for those optimally controlled) had an adjusted hazard 54% lower than the hazard for those whose HbA1c test did not adhere to the SEMDSA guidelines (*p* = 0.02) (Table 5).

## Discussion

In this study, we aimed to determine the proportions of T2DM CCMDD-enrolled patients achieving optimal glycaemic control and factors associated with an increased hazard for developing sub-optimal control. We found that only 27% of CCMDD-enrolled patients in eThekwini had optimal glycaemic control at their first HbA1c test in the study period; and that those receiving dual therapy had an increased risk, while those who adhered to HbA1c testing frequency guidelines had a lower risk. Those attending hospitals initially had a higher hazard; however, this effect decreased over time, resulting in a combined lower hazard compared to those attending PHC facilities.

Our finding of 27% of CCMDD-enrolled patients in eThekwini Metropolitan Municipality, KZN province, SA, having optimal control at their baseline HbA1c test result, was similar to findings from a cross-sectional file audit of PHC facilities in the City of Tshwane Metropolitan Municipality, Gauteng province, SA, conducted in 2019, that revealed that only 29% of CCMDD-enrolled patients had optimal glycaemic control.^16^ While our finding is congruent with this past study of CCMDD-enrolled T2DM patients in a Metropolitan Municipality in a different province of SA, we expected more patients to be optimally controlled at their HbA1c reading closest to CCMDD enrolment; as patients should have achieved a stable condition (based on two consecutive normal FPG tests) to qualify for enrolment.^12^

A reason for this discrepancy could be our study’s use of HbA1c to define glycaemic control versus the use of FPG by the CCMDD. We used HbA1c based on the IDF recommending HbA1c as the gold standard for monitoring glycaemic control.^2^ However, past studies have found HbA1c and FPG to be incongruent.^7^ The use of FPG, by the CCMDD, may be creating a false stable target group.

An additional reason may be the time interval between the first available HbA1c test and the patient’s CCMDD enrolment date. The median interval was 3 months, making it valid as a baseline measure. However, there was wide variation in the timing of the HbA1c test relative to the CCMDD enrolment date (95% CI: 13 months prior – 10 months’ post). Nevertheless, our finding is concerning and highlights an unmet need in the care cascade, that must be addressed to meet targets set out in SA’s NSP (i.e. 50% of those receiving treatment achieving glycaemic control by 2030).^9^

We also found that adherence to the SEMDSA guidelines for HbA1c testing frequency was poor in this cohort. Firstly, over the 3-year study period, 81% of T2DM patients had not taken HbA1c tests. Secondly, when looking at those optimally controlled at their first reading, 32% (695/2147) of patients had repeat test/s in the study period. This finding, is in agreement with a study of NHLS HbA1c data, from Gauteng Province (2015–2018), where 21% of patients with a first-ever HbA1c test in the NHLS, who were optimally controlled, had a follow-up result/s over their 4-year study period.^19^ These results are concerning as the STG for T2DM recommends that patients receive a HbA1c test annually, or 6 monthly, when changes are made to medications prescribed. It is worth noting, that 33% (230/695) of the cohort were enrolled in the CCMDD for ≤6 months. However, because of the 6-month window we included for HbA1c tests performed on either side of the study period, it is unlikely that many repeat tests were missed.

Thirdly, for those with repeat tests, the median interval between tests was 12 months for those that were optimally controlled and 13 months for those who were sub-optimally controlled. This is not aligned with the SEMDSA guidelines of 6 months for those optimally controlled, and 3 months for those sub-optimally controlled.^3^ It is noteworthy that the CCMDD changed its activities during the COVID-19 pandemic, so that prescription collections were extended from two-monthly to three-monthly, and clinic visits were extended from six-monthly to annually.^20^ We hypothesised that the resultant extended time between clinical check-ups may have contributed to the longer interval between HbA1c tests over this lockdown period; however, our results revealed similar time intervals between tests in periods of higher (i.e. level 4–5) lockdowns and periods with lower levels of lock down (i.e. levels 0–3).

Lastly, adherence to SEMDSA guidelines was significantly protective against developing sub-optimal control in those who began optimally controlled (aHR = 0.463, 95% CI: 0.237 – 0.905, *p* = 0.024). Correspondingly, adherence to HbA1c testing guidelines for T2DM patients has been demonstrated internationally. An Australian study, monitoring longitudinal HbA1c values in T2DM patients visiting a general practitioner (2013–2018), found that adherence to the Australian guidelines for HbA1c testing intervals, which are in line with the SEMDSA guidelines, resulted in patients remaining controlled over time. However, low adherence to guidelines resulted in increased HbA1c readings, sub-optimal glycaemic control, and higher risk for chronic kidney disease.^21^ Similarly, a United Kingdom (UK) study compared glycaemic control in patients receiving three-monthly testing to those receiving annual testing, using data from clinical laboratories, and determined that three-monthly testing was associated with a 3.8% reduction in HbA1c versus annual testing that resulted in a 1.5% increase in HbA1c.^22^

This finding demonstrates that despite the policies for care being in place, the recommended policy is not being sufficiently implemented within the CCMDD cohort. While bi-annual clinical follow-ups are appropriate and fall within SEMDSA guidelines, for those who are optimally controlled, it is not appropriate for those who are sub-optimally controlled. These patients require intensification of their clinical visits and HbA1c testing to at least as frequently as every 3 months. As it stands, many opportunities to intervene, educate, and improve glycaemic control and T2DM-related health outcomes for PLWT2DM in eThekwini, are being missed.

We also found that patients who were prescribed dual-therapy were more vulnerable to developing sub-optimal control. This concurs with results obtained in a study on the determinants of glycaemic control for PLWT2DM in Lebanon, where those using dual-therapy compared to monotherapy, had twice the odds of being uncontrolled (OR: 2.35, 95% CI: 1.58–3.50).^23^ This result could be attributed to dual-therapy being used only for patients who failed to attain optimal glycaemic control with metformin alone. As such, this group may reflect individuals with higher rates of past failures or vulnerabilities to achieving glycaemic control, before obtaining a stable status for CCMDD enrolment.

Moreover, we found an increased rate of developing sub-optimal control over time, for those attending PHC facilities compared to those attending hospitals. This result agrees with a study of NHLS HbA1c data, from Gauteng province, between 2015 and 2018, where those with sub-optimal glycaemic control, who attended hospitals had a higher likelihood of achieving optimal control compared to those attending PHC facilities.^19^ This may be explained by reports from PHC facilities of under-resourcing,^24,25,26,27^ lack of equipment,^27^ lack of infrastructure,^26,27^ and poor willingness of healthcare providers to adapt and integrate diabetes care into their service provision in SA.^26^

### Recommendations

To address the high proportion of sub-optimally controlled patients enrolled in the CCMDD, we recommend that the CCMDD consider amending their enrolment criterion to two consecutive HbA1c tests, rather than FPG tests. Additionally, including a self-efficacy score as an additional enrolment criterion may improve survival rates, especially among those receiving dual-therapy to manage their conditions. These recommendations agree with recommendations made by Ngassa Piotie et al. following their Tshwane PHC facilities file audit of CCMDD-enrolled patients.^16^ Future studies may also consider comparing glycaemic control for a CCMDD cohort using both FPG and HbA1c test results.

We also recommend that a feedback loop needs to exist, between the CCMDD and either the NHLS or the patient’s clinical results at the facility-level, so they can monitor the enrolled patient’s HbA1c. In this way, patients who change state from optimal to sub-optimal can be flagged to exit the programme. This is critical in the context of diabetes, which is a progressive disease by nature. As the disease progresses, medication management (i.e. adding insulin to the regime) and the prevention and/or management of T2DM-related comorbidities must be escalated accordingly. If there is no feedback of glycaemic control status to the programme, clinical and patient inertia may well be compounded for enrolled patients who become sub-optimally controlled.

Additionally, in SA’s resource-constrained public health sector, we recommend that the programme consider more frequent HbA1c monitoring of their patients using community health workers,^28,29,30^ T2DM education/support groups within communities,^31^ or mobile health (mHealth) technology (e.g. for patients waiting in CCMDD queues) and innovations to support self-management.^32^ In fact, our results suggest that CCMDD-enrolled patients may be better managed if point of care (PoC) testing (i.e. HbA1c or FPG) and management are built into the programme at the pick-up points, rather than at already over-burdened facilities. Such community-based interventions would offload already overburdened PHC facilities. These approaches are aligned with existing policies in SA, such as the Primary Health Care Re-engineering policy and Integrated Chronic Disease Management approach. These policies seek to mobilise, build on existing skills, and extend the scope of practice for community health workers, historically used for HIV and AIDS home-based management, to deliver NCD services, such as screening and patient-education.^28,33^ Local and international reports indicate that savings incurred from treating T2DM complications would offset costs to implement such programmes^34,35,36^; however, this would require a detailed cost–benefit analysis of each recommended approach.

Furthermore, healthcare workers should be trained in the guidelines for care for PLWT2DM, with an emphasis on the critical nature of achieving stable, optimal glycaemic control for this population. Previous studies have revealed that knowledge of the SEMDSA and STG guidelines among healthcare workers in PHC facilities is poor.^24^

Additionally, to determine why such few HbA1c tests were conducted for the CCMDD cohort, over the study period, facility-based audits and further qualitative studies are needed to establish if barriers to care exist for HbA1c testing at eThekwini hospitals and PHC facilities alike. Additionally, future studies should consider matching HbA1c results using more advanced probabilistic matching techniques. As the CCMDD does not dispense insulin, the insulin-using PLWT2DM population in eThekwini is not represented in this study. We would recommend further studies to determine the unmet need in the cascades of care for important portion of the population of PLWT2DM.

### Study limitations and strengths

There were some limitations to consider in this study. There were limited HbA1c test results available in the CDW for the study time period; thus, the survival analysis results may need to be interpreted with caution. Also, information on factors known to be associated with sub-optimal control in PLWT2DM was not available in either dataset. As a result, race, other comorbid conditions (i.e. tuberculosis), obesity measures (weight, height, body mass index [BMI]), lifestyle factors (i.e. smoking, alcohol use, diet, physical activity indices), self-efficacy, and socioeconomic factors were not included. Notably, as diabetes is a progressive condition, duration of diabetes (rather than age) is an important predictor for sub-optimal control, and the development of T2DM-related complications; however, it was not available in either data source. These unavailable variables may represent unaccounted for confounding or modifying effects, which may have distorted our results.

Despite these limitations, our study’s strengths included using retrospective data from T2DM patients spread across 13 hospitals and 107 PHC facilities in eThekwini, thus suggesting a good representation of the general CCMDD-enrolled population in eThekwini. This was also the first study involving CCMDD-enrolled patients in eThekwini. Despite the CCMDD being a large public health programme, this was the first time CCMDD-enrolled patients were linked to their clinical HbA1c results, to monitor and evaluate glycaemic control over time.

## Conclusion

While the CCMDD is invaluable in improving access to medication for PLWT2DM, this study can alert policy-makers, within the NDoH and CCMDD, that significant barriers to care still exist, as the majority of CCMDD patients are not achieving optimal glycaemic control. Addressing pertinent issues, such as reassessing enrolment criteria and creating exit criteria for those who become sub-optimally controlled, are critical. In order to flag sub-optimal cases, a mechanism needs to be established for the frequent feedback of HbA1c values into the CCMDD. Furthermore, in SA’s resource-constrained public health environment, innovative technology and community-based interventions are needed to increase HbA1c testing frequency, to align to SEMDSA guidelines, for this cohort.
